# The Mitochondria: A Target of Polyphenols in the Treatment of Diabetic Cardiomyopathy

**DOI:** 10.3390/ijms21144962

**Published:** 2020-07-14

**Authors:** Humna Bhagani, Suzanne A. Nasser, Ali Dakroub, Ahmed F. El-Yazbi, Assaad A. Eid, Firas Kobeissy, Gianfranco Pintus, Ali H. Eid

**Affiliations:** 1 Department of Pharmacology and Toxicology, American University of Beirut, Beirut 11-0236, Lebanon; hxb04@mail.aub.edu (H.B.); ahd31@mail.aub.edu (A.D.); ae88@aub.edu.lb (A.F.E.-Y.); 2 Department of Pharmacology and Therapeutics, Beirut Arab University, Beirut 11-5020, Lebanon; san413@bau.edu.lb; 3 Department of Pharmacology, Alexandria University, Alexandria 21521, Egypt; 4 Department of Anatomy, Cell and Physiological Sciences, American University of Beirut, Beirut 11-0236, Lebanon; ae49@aub.edu.lb; 5 Department of Biochemistry and Molecular Genetics, American University of Beirut, Beirut 11-0236, Lebanon; 6 Department of Medical Laboratory Sciences, University of Sharjah, Sharjah 27272, UAE; 7 Department of Biomedical Sciences, University of Sassari, Sassari, Viale San Pietro 43, 07100 Sassari, Italy; 8 Department of Biomedical Sciences, Qatar University, Doha 2713, Qatar

**Keywords:** diabetic cardiomyopathy, polyphenols, resveratrol, autophagy

## Abstract

Diabetic cardiomyopathy (DCM) is a constellation of symptoms consisting of ventricular dysfunction and cardiomyocyte disarray in the presence of diabetes. The exact cause of this type of cardiomyopathy is still unknown; however, several processes involving the mitochondria, such as lipid and glucose metabolism, reactive oxygen species (ROS) production, apoptosis, autophagy and mitochondrial biogenesis have been implicated. In addition, polyphenols have been shown to improve the progression of diabetes. In this review, we discuss some of the mechanisms by which polyphenols, particularly resveratrol, play a role in slowing the progression of DCM. The most important intermediates by which polyphenols exert their protective effect include Bcl-2, UCP2, SIRT-1, AMPK and JNK1. Bcl-2 acts to attenuate apoptosis, UCP2 decreases oxidative stress, SIRT-1 increases mitochondrial biogenesis and decreases oxidative stress, AMPK increases autophagy, and JNK1 decreases apoptosis and increases autophagy. Our dissection of these molecular players aims to provide potential therapeutic targets for the treatment of DCM.

## 1. Introduction

Diabetic cardiomyopathy (DCM) is a multifactorial phenotype consisting of ventricular dysfunction in the absence of other cardiac risk factors such as coronary artery disease, hypertension and significant valvular disease in the presence of diabetes [1]. This dysfunction may progress into outright heart failure with preserved or reduced ejection fraction [1], and it is a major cause of morbidity and mortality in the diabetic population. The definitive cause for DCM is still unclear, yet many contributing factors have been reported. These include hyperglycemia, impaired lipid oxidation, lipid accumulation in the cardiomyocytes, deposition of advanced glycated end products and endothelial dysfunction [2]. Increased apoptosis and impaired autophagy resulting from these disturbances have been also implicated in the cardiac remodeling seen in DCM [3]. Autophagy is the process by which damaged proteins and disabled organelles including mitochondria are degraded and recycled within lysosomes [4]. The double membrane bound entity that the lysosome forms with the degeneration products is known as the autophagosome. Autophagy is a normal and necessary process within the cell, but under stressful conditions, it can become dysregulated and leads to apoptosis [4]. This process can occur due to the extensive crosstalk between apoptotic and autophagic pathways, in which the mitochondria play a central role [5].

Additionally, mitochondrial biogenesis also contributes to the pathogenesis of DCM [6]. Mitochondrial biogenesis is the process by which the cell regulates gene expression of nuclear and mitochondrial genes in order to enhance ATP production. This process is usually upregulated in times of cellular stress, such as in the diabetic state [6]. Given that the mitochondria regulate many bioenergetic processes, such as lipid and glucose metabolism [7], reactive oxygen species (ROS) production and regulation [8], apoptosis and autophagy [9], its dysregulation is unsurprisingly causal for DCM.

Polyphenols are secondary plant metabolites that have been shown to improve diabetes progression and complications through various mechanisms [10], such as improving levels of oxidative stress [11] as well as enhancing insulin sensitivity [12] and lipid metabolism [13]. Polyphenols are nutraceuticals which modulate apoptosis and oxidative stress in various cell types, albeit in a cell context-dependent manner [14,15,16,17,18]. Resveratrol is a type of polyphenol found in various plants, particularly grapes and berries, and it plays important roles in human physiology and pathophysiology [19,20,21]. Some of the important mechanisms by which resveratrol elicits its effects involves the secondary messengers, cAMP and cGMP [22,23,24]. These messengers are well known to evoke pleiotropic functions, prime of which is their cardiovascular effect [24,25,26,27,28,29,30,31,32,33,34,35,36,37].

Resveratrol has been reported to modulate the function and dynamics of mitochondria, the dysregulation of which is closely associated with DCM [38]. Thus, we aim in this review to dissect some of the mechanisms by which polyphenols, especially resveratrol, potentially exert a protective effect on the mitochondria of diabetic hearts.

## 2. Effect of Resveratrol on the Mitochondrial ROS Generation and Apoptosis Pathways

In a diabetic state, there is strong evidence of increased levels of ROS production from the mitochondria and a subsequent increase in cellular apoptosis [3]. In various studies, resveratrol has been shown to modulate the amount of ROS generation under diabetic conditions through a number of pathways, thereby attenuating the acuity of resultant apoptosis. Mitochondrial uncoupling protein 2 (UCP2), a proton transporter located in the inner mitochondrial membrane, is a key mediator in the apoptotic pathway and has the capability of ameliorating ROS generation by dissipating the mitochondrial proton gradient and mitochondrial membrane potential [39].

**Figure 1:**
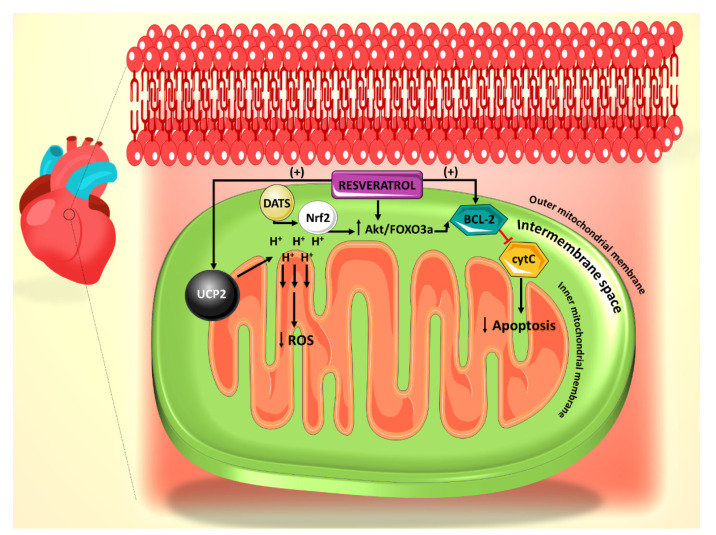
Summary of the effect of resveratrol on the mitochondrial reactive oxygen species (ROS) generation and apoptosis pathways. Resveratrol action is primarily mediated through UCP2 and Bcl-2. Diallyl trisulfide (DATS) is another polyphenol derived from garlic, which mediates its anti-apoptotic effect through Nrf2.

Increased ROS has been shown to increase the likelihood of arrhythmias [40], particularly in structurally damaged hearts with left ventricular hypertrophy such as those seen in diabetic cardiomyopathy [1]. UCP2 dysregulation leads to the alteration of mitochondrial membrane potential in this case, thus contributing to the development of arrhythmias, which may evolve to ventricular fibrillation [40]. Mitochondrial oxidative stress can also precipitate atrial fibrillation [41]. Furthermore, mitochondrial dysfunction has been reported to associate with arrhythmogenic substrates in diabetes [42]. In a recent study, it has been shown that resveratrol ameliorates cardiac dysfunction in diabetic mice via the UCP2 pathway [43]. In this regard, incubation of rat cardiomyocytes with high glucose (HG) and resveratrol, significantly increases UCP2 expression, whereas siRNA knockdown of UCP2 expression inhibits the protective effect of resveratrol. In addition, siRNA knockdown of UCP2 increases the apoptotic rate of HG/resveratrol-treated cardiomyocytes, indicating that resveratrol administration also protects against apoptosis via the UCP2 pathway [43] (Figure 1). Interestingly, the antioxidant effect of resveratrol on diabetes-induced oxidative stress has been shown to be mediated by downregulation of UCP2 expression [44]. This could be plausibly explained in the light of the fact that UCP2 is activated by ROS as a feedback mechanism [45]. With that said, it is enticing to speculate that resveratrol appears to restore the normal level of UCP2 by countering mitochondrial insult, ROS production, as it induces the antioxidant gene expression of manganese superoxide dismutase [46]. These findings also position resveratrol as a potential therapeutic agent for arrhythmias related to diabetic cardiomyopathy and other structural heart conditions.

The mitochondrial permeability transition pore (mPTP)-cytochrome c pathway is also involved in apoptosis, with cytochrome c release from the mitochondrial inner membrane being a marker for this cell death [47]. It has been shown that mPTP-cytochrome c pathway is also involved in the resveratrol-induced anti-apoptotic effect, as resveratrol treatment leads to preserved cytochrome c levels in cardiomyocytes [43]. In addition, resveratrol treatment increased the expression of B-cell lymphoma 2 (Bcl-2), a well-established anti-apoptotic factor (Figure 1).

Protective effects of resveratrol have been shown to be mediated via the dynamin-related protein 1 (Drp1) pathway, which plays a role in mitochondrial fission [48]. Increased mitochondrial fission and fragmentation occurs in response to cellular stress [49], and an interaction has been demonstrated between ROS-induced endoplasmic reticulum stress and mitochondrial fission, both of which are implicated in adipocytes damage caused by hyperglycemia [50]. In the adipocytes of mice treated with streptozotocin (STZ) to induce diabetes, increased activation of Drp1 has been observed with high glucose challenge, indicating increased tendency towards cellular stress and mitochondrial fragmentation. Administration of metformin and resveratrol prevents this effect via AMPK activation [50]. Because the Drp1 pathway has also been implicated in the cardiac response to stress and inflammation [51,52], it is possible that resveratrol administration could also decrease mitochondrial fission in a similar fashion in diabetic cardiomyocytes.

Resveratrol has also been shown to interact with the phosphoinositide 3-kinase (PI3K)/Akt/forkhead box O3a (FOXO3a) pathway in STZ-treated rats [53]. Activation of PI3K/Akt/FOXO3 pathway has been implicated in protecting cardiac cells against hyperglycemia-induced apoptosis [54,55]. Resveratrol-treated rats show dose-dependent upregulation of Akt and FOXO3a resulting in decreased number of apoptotic cells [53] (Figure 1). Moreover, resveratrol’s protective effects against HG-induced apoptosis of neonatal rat cardiomyocytes is abolished by PI3K inhibitor. Consistently, pretreatment with PI3K inhibitor results in decreased Bcl-2 expression in cardiomyocytes, indicating that PIK3 is essential for eliciting the anti-apoptotic action of resveratrol [53].

In another study [55], nuclear factor erythroid 2-related factor 2 (Nrf2), a transcription factor that modulates the antioxidant gene expression [56], has been shown to be upregulated in rat cardiomyocytes exposed to HG and treated with diallyl trisulfide (DATS), a potent antioxidant organosulfide that is found in garlic [57]. In addition, silencing of Nrf2 with siRNA abolishes DATS protection against hyperglycemia-induced apoptosis. The effect of DATS on Nrf2 has been shown to be mediated by the PI3K/Akt/Nrf2 pathway [55], consistent with other studies implicating the PI3K/Akt pathway in the anti-apoptotic effect of polyphenols [53] (Figure 1). Interestingly, a similar profile implicating Nrf2 activation in the decreased hyperglycemia-induced apoptosis in H9c2 myocardial cells has been observed upon treatment with an aza resveratrol–chalcone derivative 6b [58].

## 3. Effect of Resveratrol on the SIRT1-Dependent Mitochondrial Biogenesis Pathway

Resveratrol also plays a role in modulating mitochondrial biogenesis pathways, impairment of which has been implicated in the pathogenesis of diabetic cardiomyopathy. Sirtuin 1 (SIRT1), nuclear factor kappa B (NF-κB) p65 and peroxisome proliferator-activated receptor delta (PPAR-δ) have been found to be important molecular targets of resveratrol-induced modulation of mitochondrial biogenesis pathways.

SIRT1 is a NAD^+^-dependent deacetylase which plays a role in various mitochondrial pathways [59]. In particular, it causes the deacetylation of peroxisome proliferator-activated receptor-gamma coactivator (PGC)-1α, a transcription coactivator which plays a central role in the regulation of mitochondrial biogenesis [60]. In a recent study, resveratrol has been shown to decrease hyperglycemia-induced injury to cardiomyocytes by increasing mitochondrial biogenesis via the SIRT1-PGC-1α pathway [44]. Conversely, when SIRT1 is inhibited by sirtinol or silenced with siRNA, the protective effects of resveratrol on the mitochondrial biogenesis are abolished [44]. Additionally, SIRT1 pathway activation seems to underlie resveratrol’s protective effect on hyperglycemia-induced hypertrophy of cardiomyocytes as evidenced by the attenuated expression of the pro-hypertrophic markers atrial natriuretic peptide (ANP), brain natriuretic peptide (BNP) and β myosin heavy chain (β-MHC) [61]. In view of that, resveratrol presents itself as an important potential therapy in conjunction with other cardioprotective treatments.

**Figure 2:**
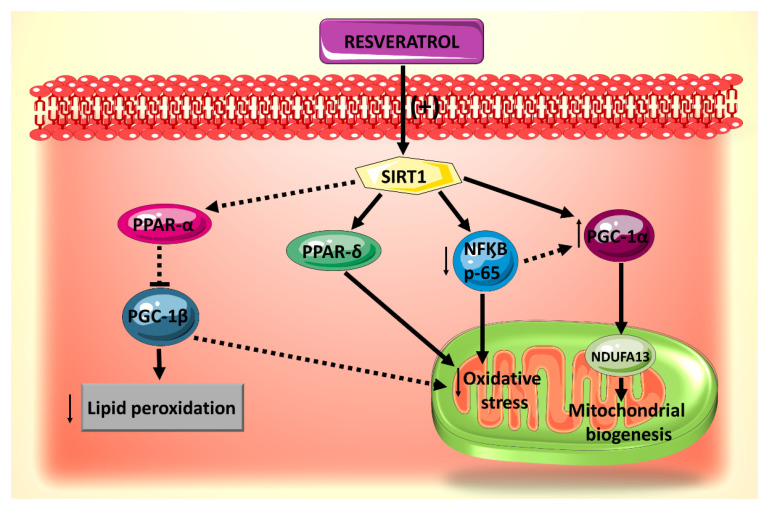
Summary of the effects of resveratrol on mitochondrial biogenesis, oxidative stress and lipid accumulation, all of which are mediated by SIRT1.

Molecular targets downstream of resveratrol activation of SIRT1 signaling pathway in mitochondrial biogenesis have been identified (Figure 2) [59]. Among these are the transcription factors PGC-1α, Nrf1, Nrf2 and a component of mitochondrial complex I, NADH: Ubiquinone Oxidoreductase Subunit A13 (NDUFA13), known as a key player in oxidative stress [62]. In this context, it has been found that pretreatment with resveratrol protects cardiomyocytes against H_2_O_2_-induced oxidative stress by upregulating the expression of the mitochondrial biogenesis-related factors in a SIRT1-dependent manner [59]. Although these findings have been observed in a non-diabetic experimental setting, it is likely to be reproducible in a DCM model, as the damage to cardiomyocyte mitochondria in diabetes is primarily mediated through the increased oxidative stress produced by increased glucose uptake and fatty acid oxidation dysregulation [63].

Another potential downstream effector of resveratrol-induced modulation of SIRT1-mediated mitochondrial biogenesis pathway is NF-κB p65. In fact, SIRT1 inhibits NF-κB signaling directly by deacetylating the p65 subunit of NF-κB complex [64]. In vivo and in vitro studies have shown that resveratrol treatment activates SIRT1 leading to deacetylation of NF-κB p65 and thereby attenuating cardiac oxidative stress and complications in diabetes [65]. On the other hand, in cardiomyocytes during inflammation, the NF-κB p65 subunit binds to the PGC-1α and blocks its activation [66]. Moreover, knocking down p65 with a specific siRNA prevents the interaction of p65 with PGC-1α and evokes a slight repercussion on PGC-1α expression [66]. In analogy, direct inhibition of NF-κB p65 by resveratrol-induced activation of SIRT1 may subsequently activate PGC-1α and modulate mitochondrial biogenesis. However, extrapolation of SIRT1/NF-κB/PGC-1α interactions to resveratrol’s protective effects against DCM warrants further investigation.

PPARδ has been shown to be involved in resveratrol-induced and SIRT1-mediated vasodilation in diabetic mice endothelial cells [67], such that when a PPARδ antagonist was administered, this vasodilatory effect of resveratrol was abolished. Activation of the PPARδ pathway is likely to be significant for resveratrol’s protective actions on the mitochondria of diabetic hearts given that PPARδ is an essential regulator of cardiac mitochondrial protection and biogenesis [68,69].

## 4. Role of Resveratrol on Mitochondrial Lipid Oxidation

Impaired lipid metabolism is a hallmark of the diabetic phenotype, and this dysregulation has been especially implicated in the development of DCM [70]. The heart uses fatty acids as its primary source of energy over glucose. However, in a diabetic state, the proportion of fatty acids to glucose usage is even higher due to insulin resistance and decreased glucose uptake [71]. The increased fatty acid uptake exceeds oxidation rates in the heart, thereby resulting in augmented accumulation of reactive lipids in cardiomyocytes and increased ROS generation due to β-oxidation of accumulated lipids [70]. It has been suggested that the surplus ROS production results in an impaired mitochondrial fatty acid oxidation as a feedback mechanism to prevent excessive reactive lipid accumulation within mitochondria [72]. Further, the mitochondrial lipid intermediates, ceramides, are known to promote apoptosis of cardiomyocytes [73]. These metabolic alterations contribute to an increased stress on the diabetic heart and ultimately lead to cardiac remodeling and dysfunction [70].

Indeed, a dysfunctional mitochondrial oxidation of palmitoyl CoA, a major lipid source available in vivo, has been observed before overt diabetes and cardiac dysfunction manifested in Zucker diabetic fatty rat models. Furthermore, accumulation of reactive lipids, increased mitochondrial ROS emission rates and elevated levels of ceramide have been also elicited in these animal models [72]. Supplementation with resveratrol improves the impaired mitochondrial respiratory sensitivity to palmitoyl CoA, ameliorates the abnormally high levels of ROS and normalizes ceramides levels and reactive lipid accumulation. Taken together, these findings point towards an important role for resveratrol as a potential regulator of the lipid dysfunction seen in DCM and present an interesting possibility to the use of polyphenol in prediabetic patients for potential protection against impaired lipid oxidation.

At the molecular level, the beneficial effects of resveratrol against mitochondrial lipid peroxidation could be mediated via the PGC-1β/PPARα. PPARα interacts with promoter regions of genes (CD36 and PDK4) that control fatty acid transport and oxidation [74]. PGC-1β is a co-activator that is recruited to the promoter regions of these genes via PPARα [75]. It has been found that PGC-1β is significantly overexpressed by high palmitate (PA) and contributes to palmitate deleterious effects, such as cardiac lipotoxicity, enhanced fatty acid uptake and oxidation, cardiomyocyte apoptosis and cardiac dysfunction [75]. Given the similarity of function between PGC-1α and PGC-1β in the context of biogenesis [44,76], and the fact that SIRT1 interacts with PPARα in the setting of cardiac metabolic dysregulation [77], and that resveratrol exerts many of its beneficial effects through SIRT1 [44,65,67], it is tempting to speculate that the SIRT1/PPARα/PGC-1β pathway is involved in the cardioprotective effects of resveratrol against DCM.

## 5. Role of Polyphenols in Autophagy–Apoptosis Interactions and Their Role in Diabetic Cardiomyopathy

The role of autophagy and apoptosis in cellular stress, particularly that resulting from diabetes and cardiovascular dysfunction, has been well elucidated [3,78]. In general, it has been shown that autophagy in cardiomyocytes is suppressed in the diabetic state [79], whereas apoptosis is highly active [3]. These two cellular processes, although seem discrete, are interrelated with each other. Autophagy dysregulation leads to accumulation of damaged mitochondria and increased mitochondrial membrane permeabilization, resulting in the release of pro-apoptotic proteins, such as cytochrome c, which can activate caspase-mediated apoptosis [80]. Moreover, they are regulated by some common signaling pathways, thus exhibiting a significant amount of crosstalk [81].

**Figure 3:**
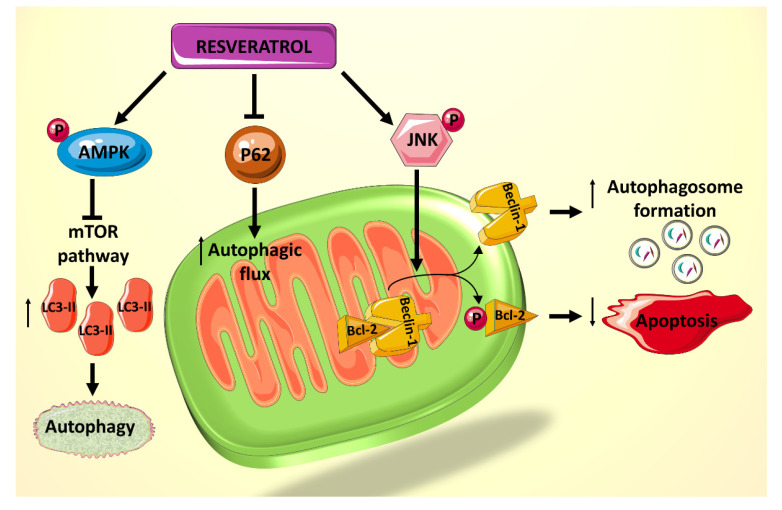
Summary of the effects of resveratrol on autophagy and apoptosis, mediated primarily by JNK1 and AMPK.

A recent study showed that rat cardiomyocytes cultured in palmitate (PA) and high D-glucose (HG) medium, mimicking that of a diabetic heart, exhibit suppressed autophagy, made evident by the decreased lipidated protein microtubule-associated protein 1A/1B-light chain 3 2 (LC3-II) and the elevated p62 levels, which are believed to be involved in autophagosome membrane expansion and autophagic flux, respectively [82]. Treatment of cardiomyocytes exposed to HG/PA with resveratrol results in p62 downregulation and LC3-II upregulation, indicating increased autophagic flux and enhanced autophagy [82]. Resveratrol treatment also decreases the amount of apoptosis induced by the HG/PA conditions. These two actions of resveratrol on the cells have been shown to be linked, as the autophagy inhibitor 3-methylademine (3-MA) diminishes RES’s effect on both processes [82]. Moreover, it has been shown that resveratrol exerts its effect on these processes via increasing phosphorylation of AMPK, thereby inhibiting the mTOR pathway and consequently increasing levels of LC3-II (Figure 3). In addition, resveratrol actions seem to mediated by an increased phosphorylation of c-Jun *N*-terminal kinase 1 (JNK1), which normally regulates the interaction between Beclin 1 and Bcl-2 [82]. Beclin 1 is a modulator of autophagosome formation [83], and Bcl-2 is a key anti-apoptotic factor [43]. When Bcl-2 binds to Beclin 1, they inhibit each other’s functions and prevent autophagosome formation and apoptosis inhibition. JNK1 phosphorylates Bcl-2 [84], thereby interrupting the interaction between the two proteins and preserving their respective activities [85] (Figure 3). Given that resveratrol increases JNK1 phosphorylation and thus Bcl-2 phosphorylation, its net effect is to increase autophagy and decrease apoptosis in HG/PA-treated cells [82]. Taken together, these results indicate a potential role for polyphenol use in vivo to reduce the deleterious effects of autophagy and apoptosis dysregulation on DCM.

Another study focused on the effect of tea polyphenols (TP) on autophagy in obese (OB) rats with DCM induced by STZ [86]. It has been found that in OB rats (without a diabetic phenotype) the levels of LC3-II and Beclin 1 are decreased whereas p62 levels are increased, indicating decreased autophagy and decreased flux, respectively. These effects have been prevented with TP treatment. Phenotypically, the OB rats showed irregular myofilaments and disordered mitochondria, whereas the TP-treated OB rats showed normal myofilaments and mitochondria with some autophagosome formation. These results are striking as they contradict other reported study [82] as well as the general understanding that decreased autophagy is a precipitating factor in DCM.

It is plausible that a greatly increased level of apoptosis in DCM is the source of the autophagy dysregulation, and perhaps the coupling of the two processes fluctuates throughout the progression of the disease based on the level of cellular products in need of degeneration and recycling. Nonetheless, tea polyphenols have been shown to ameliorate the phenotype of DCM, including the visualized mitochondrial disturbances. This further supports the potential therapeutic benefit of polyphenols on cardiac dysfunction in diabetes.

## 6. Conclusions

There is undeniable evidence that polyphenols, particularly RES, play a significant role in hampering the progression and prognosis of DCM in animal models. They target multiple pathways that are central to the mitochondrial dysfunction associated with DCM and ameliorate the effects of a HG and lipid environment. These findings lay a strong foundation for future clinical investigations, and it would also be interesting to explore the effects of targeted resveratrol treatment on endoplasmic reticulum stress and its interaction with autophagy.
